# Feasibility and preliminary efficacy of a school-based health and well-being program for adolescent girls

**DOI:** 10.1186/s40814-021-00964-3

**Published:** 2022-01-22

**Authors:** Karen White, David R. Lubans, Narelle Eather

**Affiliations:** 1 Priority Research Centre for Physical Activity and Nutrition, University of Newcastle, Callaghan, New South Wales Australia; 2 Faculty of Education and Arts, University of Newcastle, Callaghan, New South Wales Australia

**Keywords:** Adolescent, Female, Well-being, Mindfulness, Physical activity, Screen-time

## Abstract

**Background:**

The primary aim of this pilot study was to evaluate the feasibility and preliminary efficacy of a school-based health and well-being program (Health and Well-being for Girls: HWBG) on adolescent girls’ well-being, mindfulness, screen-time, and physical activity**.**

**Method:**

The HWBG program was evaluated using a group randomized controlled trial (RCT) in one secondary school. A convenience sample of female students in Grade 8 (mean age 14 ± 0.5 years) was recruited for the study (*n*=4 classes; 89 girls). The 20-week multi-component HWBG program, guided by Self-Determination Theory and Acceptance and Commitment Therapy, was designed and delivered by a member of the research team. Randomization occurred prior to baseline assessments at the class level (*n*=4 classes) into dose-matched treatment conditions (i.e., two classes received the HWBG intervention and two classes received an alternate elective course as a wait-list control group). Process evaluation measures of recruitment, retention, adherence, and satisfaction were used to determine program feasibility. This study was designed to assess feasibility (primary outcome) and preliminary efficacy (secondary outcomes), rather than effectiveness. Preliminary efficacy of HWBG for improving mental and social health, screen-time, and physical activity were measured and analyzed using linear mixed-models.

**Results:**

“The health and well-being program” targeting adolescent girls was found to be feasible for implementation as an elective course in a secondary school setting. Participants in the HWBG program reported high levels of satisfaction, the majority of lessons (45 out of 50), were implemented as planned and adherence to the planned HWBG program content was very high. Medium positive effects on mental health (*d*=0.45) and social health (*d*=0.50) were observed. Small effects were observed for physical activity, and no significant intervention effects were found for levels of recreational screen time. Participant ratings showed high levels of perceived benefit and enjoyment as indicated in the evaluation data (mean rating of 4.44 out of a possible 5.0) when asked whether the program was enjoyable.

**Conclusion:**

The study provides preliminary support for HWBG as a potentially feasible and enjoyable program suitable for use with adolescent girls in the secondary school setting. Potential of the study for facilitating improvements in pro-social and psychological well-being is also supported.

**Trial registration:**

ACTRN12617000157370. The trial was registered with the Australian New Zealand Clinical Trials Registry.

## Key messages regarding feasibility

1. What uncertainties existed regarding the feasibility?
  The Health and Well-being for Girls (HWBG) program was a feasible approach when delivered by a member of the research team in one secondary school. However, the feasibility of a “train the teacher model” is yet to be determined. Additional mindfulness training for teachers of HWBG may be required to ensure equally successful outcomes.
2. What are the key feasibility findings?
  A health and well-being program targeting adolescent girls is feasible for implementation as an elective course in one secondary school setting. Participants in the HWBG program reported high levels of satisfaction. All lessons were implemented as planned, and adherence to the HWBG program content was very high when delivered as a curriculum program.
3. What are the implications of the feasibility findings for the design of the main study?

Findings from the study will be used to refine HWBG before progressing to a cluster FRCT involving multiple schools. Implications about the feasibility of such a high dose (e.g., full elective—5 h per fortnight) or modification to make the program available for all girls in a different format.

## Introduction

Data suggest that approximately 20% of adolescents worldwide have a diagnosed mental health illness [30, 106]. These rates are considered an underrepresentation, as many health disorders in adolescents go unreported, and treatment options are not accessed by up to two thirds of young people with depression and anxiety [3, 106]. Levels of stress, anxiety, bullying, body image concerns, insomnia, and depression are increasing among adolescents globally [105]. In Australia, over 40% of respondents in The Mission Australia Youth Survey report (2017) indicated that they were either extremely concerned or very concerned about coping with stress, and 20% of respondents reported being either extremely concerned or very concerned about depression [16]. Of particular concern is the recent rise in levels of mental health difficulties in adolescent girls [43].

The World Health Organization defines mental health as “a state of well-being in which the individual realizes his or her own abilities, can cope with the normal stresses of life, can work productively and fruitfully, and is able to make a contribution to his or her community” ([34], p. 231). Mental health includes both positive and negative indicators [86], with research demonstrating that adolescent girls fare much worse than adolescent boys in measures of self-concept, life satisfaction, negative feelings, and self-harming [32, 43]. From 2012 to 2015, there was a 50% increase in the reporting of depressive symptoms and self-harm in Australian adolescent girls, with total rates of depression and self-harm almost double those of their male peers [16]. Even though suicide rates have increased for both sexes, rates have increased threefold for girls aged 12–14 years since 2007. By comparison, the rates for boys of the same age have doubled [35, 95].

The causes of mental health disorders in youth are complex, but research supports that satisfying the need for interpersonal relationships are essential for good mental and physical health [50]. High levels of loneliness are reported among adolescents, with one in four young people disclosing “problematic” levels of loneliness [58]. Each year a rising number of Australian children contact children’s support services about feelings of isolation and loneliness, with the latest figures demonstrating that support services delivered 4636 counseling sessions for loneliness in 2017/2018 [20]. Links between loneliness and a broad range of mental health difficulties (e.g., suicidal ideation, anxiety, depression, substance misuse, and self-injury) [46, 61, 90] are also well supported. Evidence of effective interventions addressing levels of loneliness in adolescents are limited, but strategies focusing on the promotion of good social health and social connectedness may offer a level of protection for young people from loneliness ([22, 58].

“Positive Education” has emerged as a potential model to promote adolescents’ well-being [88] and has increased in popularity over the last decade [49]. Positive education brings together positive psychology science with best practice teaching, in order to support and encourage individuals to flourish within their communities [74]. Positive education has been referred to as a traditional model of education focused on the development of academic skill, complemented by approaches that promote good mental health and nurture well-being [88]. For example, positive education focuses on building specific skills that support students to build positive emotions, strengthen relationships, encourage a healthy lifestyle, promote mindfulness, and enhance personal resilience; underpinned by a focus on character strengths [74]. Review level evidence demonstrates that positive psychology interventions have had a positive impact on levels of student well-being and academic performance [99].

A crucial component of positive education and good mental health is connectedness [102], which is conceptualized as a sense of belonging, perceived sense of care and support, a sense of perceived closeness to a group or individual, and comfort in discussing problems with others [9]. Furthermore, school connectedness (the levels to which an adolescent feels accepted, respected, valued, and included in school) is an established protective factor for adolescent health, social well-being, and education [91]. Increased optimism, improved academic performance, resilience, and health promoting behaviors (such as nutrition and physical activity), along with lower levels of depression and problem behavior, are also related to higher individual levels of school connectedness [4, 13, 18, 80, 103]. The goals of positive psychology are supported in shifting from a focus on psychopathology to a greater consideration of positive functioning and identification of individual strengths [50].

Adverse mental and physical health outcomes are associated with excessive recreational screen-time (i.e., screen use for entertainment), and this is a global public health issue. Systematic reviews and meta-analyses provide evidence for a negative association between internalizing symptoms (e.g., depression and anxiety) and recreational screen-time in youth [47, 59, 108]. This is particularly strong for adolescent girls with research citing body image, online bullying, harassment, and self-esteem issues as mechanisms linking recreational screen-time to poor mental health [55, 95]. It is argued that the increase in social media use, of which adolescent girls are the primary users, has coincided with the spike observed in the rates of anxiety and depression in this age group [40, 55, 89]. Although reducing screen-time has been identified as a potential strategy to improve adolescents’ psychosocial health [93], findings from experimental studies have been mixed [8]. Studies conducted among adolescents in the USA indicate that girls aged 13–18 years are more likely than their male peers to struggle with depressive symptoms and suicide-related tendencies in relation to time spent on screen media and devices [96]. Conversely, the US adolescents engaging in activities devoid of devices such as exercise/sports, in person social interaction, homework, and print media were less likely to report these mental health issues ([96]).

Physical activity is another lifestyle behavior that is positively associated with mental health in adolescents [8]. However, given that only 18% of Australian adolescents (12–17 years) participate in the daily recommendation of 60 min of moderate to vigorous physical activity [1] and spend over 2 h a day (136 min) engaged in screen-based activity, most young people spend more time on screens than they do moving [6]. There is strong evidence that regular physical activity can improve physiological outcomes and health-related fitness in children and adolescents [8]. Evidence also supports that physical activity can improve adolescents’ mental health [7, 11, 12] via a range of neurobiological, psychosocial, and behavioral mechanisms [8].

In comparison to those that are active, inactive adolescents demonstrate reduced levels of well-being and increased levels of internalizing problems (e.g., anxiety and depression) [66]. Adolescent females are less active than adolescent males [5], and interventions targeting adolescent girls have had limited impact on objectively measured physical activity [8]. Interventions underpinned by theories of behavior change, and those that have a multi-component design, designed for girls only and delivered in schools, have proven to be the most effective approaches to improving physical activity levels in adolescent girls [77, 79]. In addition, interventions have been found to be more effective when the enjoyment of the activity within the lesson has been prioritized and when adolescent girls are provided with autonomy in lessons (e.g., input into activity, partner/teams, or rules) [17].

Interventions that facilitate the development and maintenance of meaningful relationships can also produce long-term psychological benefits for adolescents [41, 51]. Despite this evidence, programs targeting adolescents that address the recent and increasing prevalence of mental health disorders are lacking [78, 100]. Health and Well-being for Girls (HWBG) is a unique program that is the first of its kind to address levels of mental health and well-being, mindfulness, physical activity levels, and screen-time behaviors into one program, with a theoretical grounding in Self-Determination Theory and elements of Acceptance and Commitment Therapy. Therefore, the primary aim of this study is to evaluate the feasibility of a school-based health and well-being intervention on adolescent girls’ levels of well-being, mindfulness, physical activity, and screen-time.

## Methods

### Design

The (HWBG) program was evaluated using a group randomized controlled trial (RCT) in one secondary school. Baseline assessments were conducted in February 2017 and were repeated at a 6-month follow-up (June 2017 immediate post-intervention). The design, conduct, and reporting of this study adhere to the Consolidated Standards of Reporting Trials (CONSORT) guidelines [29] and utilized the extension for Pilot and Feasibility Studies version of the CONSORT guidelines. Ethics approval for the study were obtained from the University of Newcastle, Australia (Approval No: H-2015-0189) and the New South Wales (NSW) Department of Education and Training human research ethics committees (SERAP) 2016644. The trial was registered with the Australian New Zealand Clinical Trials Registry**:** ACTRN12617000157370. Our results should be interpreted with caution. This study was not adequately powered to detect changes in any of the secondary outcomes. Alternatively, this study involved a convenience sample to evaluate the feasibility of the intervention when offered as an elective course to adolescent girls in year 8. Secondary outcomes were assessed to determine evidence of promise for improvements resulting from the intervention.

### Participants

A member of the research team explained the program to the school principal, teachers, and students prior to recruitment. All participants (i.e., principal, teachers, and students) were required to provide written informed consent. All Grade 8 girls were eligible to participate in the study unless they had a medical condition preventing assessment or participation in the program, and those with signed consent forms were included. Randomization occurred at the class level (*n*=4 classes) into dose-matched treatment conditions (i.e., HWBG or elective course) prior to baseline assessments. An independent researcher randomized the classes using a computer-based random number-producing algorithm. This approach ensured an equal chance of allocation to each group. Randomization occurred prior to baseline assessments, with assessors only blinded at baseline. The sample recruited for this study was a convenience sample to evaluate the feasibility of the intervention when offered as an elective course to adolescent girls in year 8. Secondary outcomes were assessed to provide insights into the effect of the program and to determine the suitability of the measures. A sample target of approximately 90 students was recruited (four classes of approximately 22 students).

### Intervention

#### Intervention conditions

The HWBG program was guided by Self-Determination Theory [24] and aligned with concepts from the Acceptance and Commitment Therapy [45]. The program was delivered during 5 h of timetabled curriculum time per fortnight over two school terms (20 weeks). Theoretical learning activities (detailed in Table 1) targeted concepts of body image, self-acceptance, mindfulness, self-compassion, stages of girlhood, celebrating differences, recognizing strengths, cyber safety, decision making, leadership, issues surrounding consent to varying lifestyle factors, consumerism, identifying quality relationships, gratitude, trust, compassion, and developing empathy. Practical lessons included mindfulness training and meditation, yoga, Pilates, games for fun, and fitness activities. An experienced and qualified physical education teacher (lead researcher), designed, and delivered the HWBG program.

**Table 1:** Health and Well-being for Girls Program Components (Australia 2020)

	Health and Well-being Program for Girls (HWBG)	
**Components and content**	**Weeks**	**Alignment of content with SDT and ACT**
**Physical activity**		(SDT) Autonomy targeted by allowing girls to choose the program initially and then the level of participation in lesson activities. Development of skill competence to participate in physical activities relevant to them in a supportive environment, transcending competition and judgment. (ACT) Being present, focusing on the here and now. Diffusion of thoughts and self as context—unchanged by time and experiences
• Yoga	1–4	
• Pilates	5	
• Fitness	6–10	
• Walking	8–9	
• Games for fun and competence	11–20	
**Mindfulness practice** Meditation /Breathing Techniques Attitude of N.A.C.K (*The Mindfulness Clinic)* Recognizing emotion	1–20	(ACT) Being present/diffusion of thoughts and unchanged by experiences. (SDT) Relationships with self and others.
**Introduction to HWBG**	1	(SDT) Develop competence to recognize and acknowledge differences and similarities between peers. How is this program relevant to me as a girl?
**Goals of physical activity in HWBG** Difference between physical appearance and physical ability. Australian and UK Government initiatives for participation in physical activity.	1	(ACT) Diffusion of thoughts and self as context–—unchanged by time and experience. (ACT & SDT) Discussions relating to trusting our instincts and developing the courage and autonomy to choose one’s own behaviors and make independent decisions relating to health issues. (ACT) Diffusion of thoughts, self as context—unchanged by time and experience. Acceptance of difficult thoughts and emotion. Present in the here and now. (SDT) Close, affectionate relationships with self and others.
**Attitude of mindfulness** • NACK and ABCD *(‘The Mindfulness Clinic’)*	2	
**Self-acceptance activities** Celebrating our unique differences and imperfections. Recognising our similarities as human beings and promotion of connectedness.	3–4	Acceptance of challenges as part of life. Identification of similarities and differences among human beings. (SDT) Relatedness to the challenges facing adolescents and close relationships with self and others. (ACT) Values/Acceptance and Commitment. What does it mean to be a girl? Self as context, unchanged by time and experience.
**Creation of butterfly**	5	
**Self-compassion** The difference between self-esteem and self-compassion.	6	(SDT) Competence to recognize how to be supportive of the self when experiencing personal challenges. Competence to lead and the autonomy to embrace our challenges. (ACT) Acceptance of difficult emotion and commitment to valued action.
**Mindful listening and mindful eating** Mindfulness practice	7	(SDT) Competence to listen to another’s point of view and being present. Close and affectionate relationships with others. (ACT) Observing thoughts without being ruled by them.
**Leading with bravery and courage** Identifying Strengths and Embracing Vulnerabilities	7	(SDT) Relatedness to challenges facing adolescent girls and competency to cope through character strength identification. (ACT) Commitment to pursue the important things in their lives.
**Managing emotion in life and relationships.**	8	(SDT) Competence to recognize and manage challenging emotions and autonomy to choose how to respond. Relatedness to everyday life. (ACT) Diffusion of thoughts and commitment to valued action.
**Girlhood to womanhood** • Stages of girlhood (*Steve Biddulph*) • Safety • Curiosity • Getting along with others • Identifying interests/ abilities and character strengths • Identifying needs and wants in relationships • Qualities of good relationships • Preparation for adulthood	9–10 9–10	(SDT) Relatedness to adolescent challenges and competence to make informed decisions. Autonomy to act in a safe manner. (ACT) Values and commitment (SDT) Relatedness to adolescent girls in forming quality relationships through life and relative to pressure in adolescence. Autonomy to identify strengths, interests, and the competence to pursue these throughout adolescence.
**Consent to sexual activity** “Too sexy too soon”—How happy sexuality grows by itself (*Steve Biddulph & Danielle Miller*) “Kaysee & Genevieve”—*Steve Biddulph* Drugs and their effect on decision making	11	(ACT) Values and commitment to pursue quality relationships of individual choice. (SDT) Autonomy and competence to make a personal choice related to values.
**Sexual health and personal value** Putting on a male condom (Family Planning NSW)	11	(SDT) Autonomy to value the self in difficult situations throughout adolescence and competence to choose wisely. (ACT) Presence and valuing what is truly important with commitment to personal values.
**Cyber awareness and cyberbullying** Cybersafety and behavior online	12	Relatedness to current experience in the twenty-first century adolescence and the autonomy to choose behaviors in regards to screen time. Competence and self-regulation developed through mindfulness and meditation. (ACT) Acceptance, values, diffusion, and being present. (SDT) Relatedness to adolescent girls and autonomy to choose what is important to them. Autonomy to choose ethically in accordance with values and morals. Resistance to peer pressure. (ACT) Values & Commitment. Being present and accepting.
**Screen time, social media, and well-being** Impact of screen time on adolescent well-being “White space” and the importance of boredom.	13	
**Overcoming adversity/mentoring** Mentor Task—Bethany Hamilton	13	(SDT) Competence and autonomy to resist peer pressure and make personal decisions in alignment with individual goals and interests.
**Gratitude and the human spirit** Gratitude Assessment Task “365 Grateful”	14	(SDT) Relatedness to life during adolescence. Competence to identify elements in life to be grateful for and commitment to value those elements (ACT).
**Consumerism—“The Big Sell”** “Too Much Too Soon”—Stolen Childhoods “Ruby Who & Project Girl”	15	(ACT) Diffusion of thoughts and self as context—unchanged by time or experience. Being present and acceptance of difficult thoughts and emotions. Value and commitment. (SDT) Autonomy to choose and decipher what is important in life.
**Body image and body confidence** • The Trickery of Media/Unrealistic Ideals • The Body Image Movement—Taryn Brumfit • Body image problems posed by Social Media • Fabricating Beauty—Butterfly Foundation • “Dove—Confident Me” Workshop • Dear Body—Kindness and Compassion to the Body (*The Mindfulness Clinic*)	16–18 16–18	(SDT) Competence to decipher what is unrealistic and unachievable in relation to body image ideals. (ACT) Acceptance and allowance of difficult thoughts and emotions when feeling inadequate in regards to the ideal body image projection. Unchanged by experiences of images viewed on social media.
**Relationships and connectedness** Butterfly Ribbon Activity (*The Butterfly Foundation*) Warm fuzzies activity	19–20	(SDT) Relatedness to personal relationships and consolidating social connections among the group. (ACT) Commitment to maintaining positive relationships and valuing connections.

*SDT* self-determination theory, *ACT* acceptance and commitment theory

#### Control conditions

The wait-list control group participated in an elective subject of their choice (dose-matched). The elective subjects were provided for 5 h per fortnight, over two school terms (20 weeks) and varied considerably. Subject choices ranged from technology and science through to performing and creative arts. The wait-list control group received the intervention in full; however, evaluation data for the control group were not collected.

### Measures

Assessments were conducted at baseline (February 2017) and at 6 months (July 2017) by a member of the research team, who was not a teacher at the school and was blind to the treatment allocation. This member of the research team was blinded to group allocation at both time points.

### Feasibility

The feasibility of the program was examined using process evaluation measures of (i) *retention* (how many participants completed the program and participated in all assessments pre- and post-intervention), (ii) *compliance* (participants’ attendance to HWBG lessons), (iii) *adherence* (number of HWBG lessons delivered), (iv) *fidelity* (components of each lesson delivered), and (v) *satisfaction* (participants’ post program evaluation survey results). The evaluation survey was administered at the completion of the program, with 16 items assessing satisfaction and perceived benefits using a 5-point Likert scale format (responses ranging from “Strongly Disagree” = 1 through to “Strongly Agree” = 5). Participants were also asked to indicate the specific activities they enjoyed and to comment on aspects of the program they did not enjoy (open-ended format).

### Preliminary efficacy outcomes

Demographic information was collected (age, grade, and location), and a purpose-designed questionnaire based on previously used and published scales suitable for use with adolescent girls was administered. The following outcomes were assessed:

#### Psychological health

Strength and Difficulties Questionnaire (SDQ): This one-page (25-item) self-report measure of psychological adjustment. Using a 3-point Likert scale with response options of (1 = not true to 3= certainly true). The total score of each scale is used to generate scores for conduct problems, hyperactivity, emotional symptoms, peer problems, and pro-social behavior; all but the pro-social behavior are summed to generate a total difficulties score [36, 37]. The SDQ has been shown to be a reliable and valid indicator of psychopathological symptoms in adolescents [68].

#### Psychological well-being

Diener and colleagues’ psychological flourishing scale (2010) was used to measure subjective well-being. Using a 7-point Likert scale (1 = strongly disagree, to 7= strongly agree), students respond to statements relating to indicators of “eudemonic” well-being (e.g., *I lead a purposeful and meaningful life*). Items in the scale are summed to create a composite well-being score (possible range 8 to 56). The validity of the measure has been established previously [25].

#### Mindfulness

Child and Adolescent Mindfulness Measure consists of a 10-item scale using a 5-point Likert scale (ranging from “never true” to “always true”) [38]. (e.g., *I think about things that have happened in the past instead of thinking about things that are happening right now).*

#### Motivation: autonomous motivation for physical activity

Autonomous motivation for physical activity was assessed using the autonomous motivation subscale of the Behavioural Regulations in Exercise Questionnaire-2 [62]. Students responded on a 5-point Likert scale how true each statement was for them (1 = not true for me, to 5 = very true for me). Items were adapted to reflect participation in “physical activity” rather than exercise (e.g., *I value the benefits of physical activity*). The factorial validity of the measure has been established previously [62].

#### Self-compassion

Self-compassion was measured using the 26-item Self-Compassion Scale [71], which has been shown to be a valid and reliable measure of self-compassion [72]. Items are both positively and negatively worded and items use a 5-point Likert scale ranging from 1 (almost never) to 5 (almost always). (e.g., *when I am going through a hard time, I give myself the caring and tenderness I need*).

#### Rumination

Rumination was measured using the 22-item measure, which utilizes a 4-point Likert Scale. This scale has been previously adapted for use with adolescents, where the directions are modified so that participants report on their responses to feeling “stressed or upset” rather than “depressed” [94]. For each item, participants indicated how often they “think or do each one” when they “feel stressed or upset” using a 4-point scale: 1 (almost never), 2 (sometimes), 3 (often), or 4 (almost always) [92, 94].

#### Screen time

Recreational screen-time was measured using a modified version of the Adolescent Sedentary Activity Questionnaire (ASAQ) [42]. The ASAQ requires subjects to self-report the total time spent engaged in a variety of recreational screen behaviors. Total screen-time is then determined as the sum of time spent in each screen behavior. The ASAQ has excellent reliability (Cronbach’s *α* = .78 and .90 for girls and boys in Grade 8, respectively) [42] and is considered a comprehensive measure of sedentary behaviors among young people [42].

#### Social health: a modified version of the Hemingway

The measure of adolescent connectedness [54] [53] was used to measure connectedness. The subscale “self-in-the-present” was used and consists of six items using a 4-point Likert style format (ranging from 1= not true to 4 = very true).

#### Physical activity

Participants were asked to wear a sealed Yamax SW700 pedometer (Yamax Corporation, Kumamoto City, Japan) during their normal daily activities to measure physical activity for seven days (including three consecutive days and one weekend day) [85]. This is a validated objective measure of physical activity for use with young people [64]. The participants were asked to wear the pedometers at all times other than when sleeping or when they might get wet. Students recorded the step counts and then reset the pedometers at the start of the school day (9am) on Monday through to Friday during the assessment periods.

### Statistical analysis

Statistical analyses of all outcomes were conducted using linear mixed-models using IBM SPSS Statistics for Windows (Version 20) (SPSS, INC 2010, IBM Company, Armonk, NY) with alpha levels set at *p* <0.05. The mixed models will be used to assess the preliminary efficacy of treatment (HWBG or control), time (treated as categorical with levels that include baseline and immediate post-intervention follow-up), and the group-by-time interaction. Mixed-models are consistent with the intention-to-treat principle. Kolmogorov-Smirnov test was used to determine normality of data and 95% confidence intervals calculated. The mixed model analysis was used to assess the preliminary efficacy of treatment (HWBG or control), time (treated as categorical with levels that include baseline and immediate post-intervention follow-up) and the group-by-time interaction. Cohen’s *d* was calculated by dividing the mean difference in change by the pooled standard deviation of change for each variable, and interpreted as 0.2=small effect, 0.5=medium effect, and 0.8=large effect [21].

## Results

**Fig. 1:**
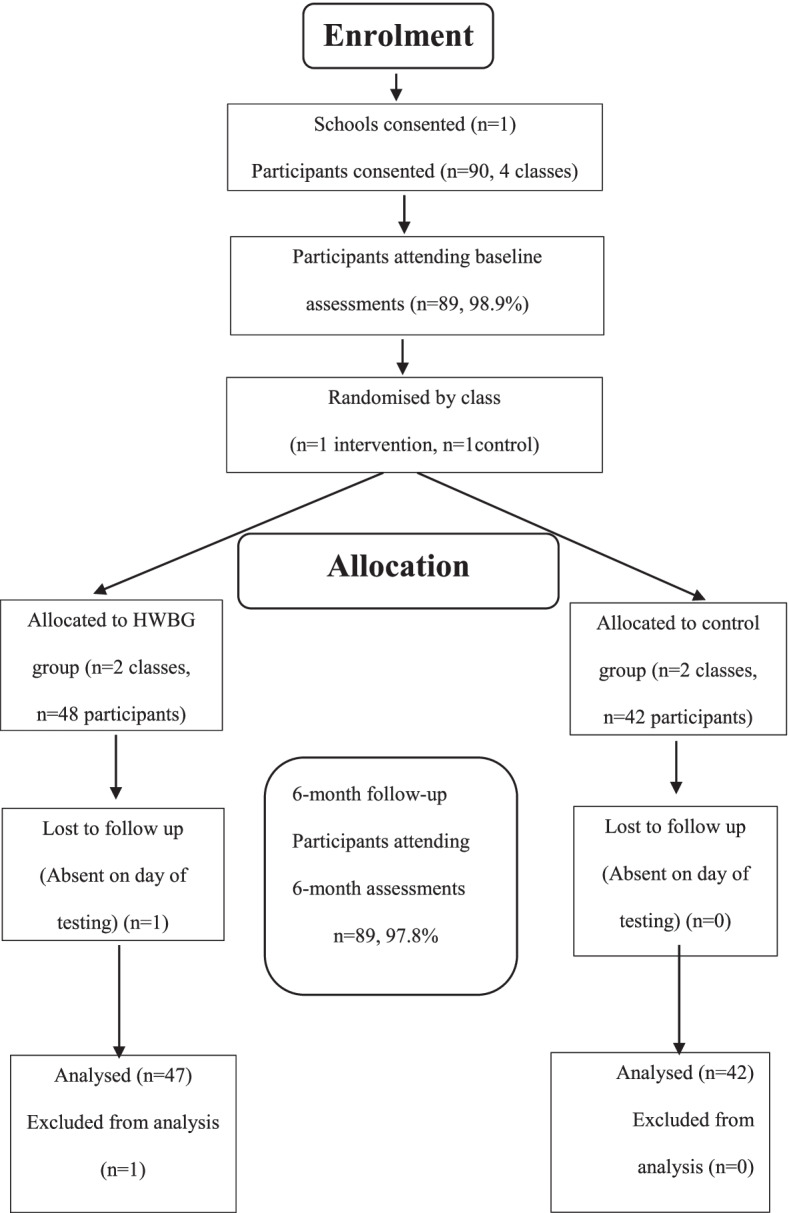
Flow of participants through the HWBG study

Table 2 details all results for primary and secondary outcomes. The flow of participants through the HWBG study is displayed in Fig. 1. Table 3 describes the characteristics of the participants. In summary, participants for the study were year 8 girls (*n*=89, mean age 14 ± 0.5) from one secondary school located in a low-income area of NSW Australia, identified using the Socio-Economic Indexes for Areas (SEIFA) Index of Relative Socio-economic Disadvantage.

**Table 2:** HWBG intervention effects (by treatment group) (Australia, 2017)

	Student outcomes										
Measure	Control group (***n*** =41)			HWBG (***n***=48 )					Adjusted difference in change (95% CI)a	Group*Time ***P*** value	Cohen’s ***d*** Effect size
	Baseline (1)	SD	6 months (2)	SD	Baseline (1)	SD	6 months (2)	SD			
Social health	16.07	3817	15.29	3378	15.33	3204	16.25	3657	1.7 (0.25–3.16)	**0.022**	0.50
Strengths & difficulties	33.20	5.972	33.83	6.221	35.37	6499	33.98	7447	−2.0 (−3.93–0.10)	**0.039**	0.45
Prosocial	12.78	1.754	12.90	1.446	12.43	2000	13.11	1574	0.6 (−0.20–1.32)	0.149	0.32
Hyper	9.51	2.135	9.54	2.303	10.25	2260	9.77	2386	−0.5 (−1.29–0.29)	0.214	0.23
Conduct	7.17	1.672	7.29	1.647	7.53	1745	7.33	2107	−0.3 (−1.03–0.38)	0.358	0.20
Peer	7.10	1.772	7.49	1.599	7.84	2230	7.23	2086	−1.0 (−1.65–0.34)	**0.003**	0.65
Emotional	9.42	2.598	9.51	2.812	9.76	2359	9.65	2840	−0.2 (−0.99–0.58)	0.610	0.11
Self-compassion	37.00	8.198	36.42	8.832	36.16	7369	37.37	6896	1.8 (−0.58–4.17)	0.136	0.32
Mindfulness	21.34	7.780	23.46	8.028	20.53	7115	23.22	9690	0.6 (−2.83–3.97)	0.741	0.07
Flourishing	46.56	5.550	44.53	6.957	45.43	6804	44.61	9162	1.2 (−1.70–4.10)	0.412	0.18
Externalizing	16.68	3.282	16.83	3.376	17.78	3513	17.10	3970	−0.8 (−2.07–0.41)	0.189	0.28
Internalizing	16.51	3.957	17.00	3.931	17.59	3796	16.88	4335	−1.2 (−2.28–0.12)	**0.030**	0.47
Rumination	22.61	6.786	22.85	7.641	23.22	7238	23.32	8069	−0.1 (−2.56–2.26)	0.903	0.02
Weekday steps	8352.69	3196.055	8611.48	2955.087	9174.65	2929.025	10252.56	5048.763	819.1 (−1181–2819)	0.414	0.07
Weekend steps	7928.56	3985.532	7605.40	3908.533	8256.02	3883.663	10,205.41	5110.090	2273 (−356–4901)	0.089	0.32
Total steps	59,562.32	21,706.257	58,526.15	21,402.426	62,775.16	19,548.277	72,922.73	32,364.013	11,184 (−3164–25,532)	0.124	0.24
Screen-time total	26.04	8.963	26.32	8771	27.68	8389	29.08	10224	1.1 (−3.0–5.3)	0.592	0.09
Weekend screen time	12.80	4.412	13.15	4.368	13.44	4731	14.73	4872	0.9 (−1.3–3.2)	0.397	0.13
Weekday screen time	13.24	5.150	13.17	4811	14.24	4607	14.38	5874	0.2 (−2.2–2.6)	0.859	0.03

**Table 3:** Descriptive characteristics of study participants

Characteristics	Total (***N*** = 89)
**Age, mean (SD), years**	Grade 8 (mean age: 14 ± 0.5 years)
**Sex**	Female
**Cultural background, n (%)**	
Australian	83 (93.3)
Other	6 (6.7)
**Language spoken at home, n (%)**	
English	87 (97.8)
Other	2 (2.2)

### Feasibility results

Information leaflets, parental, and participant consent forms were sent home with all students in Grade 8 (aged 13–14 years) who elected to complete the Well-being elective in 2017. Ninety students out of 91 students (98.9%) provided assent and written consent to participate in the study.

#### Retention

Eighty-nine out of 90 (98.9%) participants completed the program and participated in all assessments at baseline and post-test.

#### Compliance

On average, 88% of participants attended five lessons per fortnight for 20 weeks.

#### Adherence

A total of 50, 1 h lessons (five lessons per fortnight), were scheduled to occur over 20 weeks or two school terms, with three of the five lessons scheduled as theory-based lessons and two of the five to be scheduled as practical-based lessons. Forty-five lessons (90%) were conducted as planned in the timetabled spaces assigned to the participants. Public holidays and staff development days affected the full delivery of scheduled lessons by interfering with all of the five lessons missed across the 20-week program.

#### Fidelity

All 45 HWBG lessons were delivered as planned.

#### Satisfaction

Mean scores on the evaluation survey categories ranged from 3.71 to 4.48 of a possible 5 (1=Strongly disagree to 5=Strongly agree) (see Table 4), for the 16 items in the evaluation survey, indicating high to very high overall satisfaction rates for the program. “I think that this program is so important to girlhood. I think that this class should be more open to other schools. I am grateful to have been in this class and learning that it is okay to be myself”. “I loved this program, it has taught me to be kind and grateful to myself and others. I really enjoyed meditating”. “The HWBG program helped me with the issues that are occurring in society such as body shaming, stress and criticism”. “The sport has paid off and was very enjoyable”.

**Table 4:** HWBG study evaluation data

Evaluation data			
		Mean	SD
**Perceived benefits**			
1.	The HWBG program provided me with useful information about self-acceptance, self-compassion and appreciation for who I am	4.38	0.70
2.	The HWBG program provided me with useful information about managing stress and anxiety	4.46	0.74
3.	The physical activity sessions helped me to realise that I can do fitness activities	4.08	0.87
4.	The mindfulness practice provided me with useful information about managing stress	4.21	0.74
5.	The mindfulness practice helped me to manage my thoughts, emotions and ability to focus	4.10	0.66
6.	The meditations were helpful	4.19	0.70
7.	The lessons on body image helped me to recognise how images are changed and manipulated	4.38	0.82
8.	The body image lessons helped me to realise that I don’t have to be perfect	4.48	0.68
9.	The yoga activities made me feel calm	3.71	0.92
10.	Being able to listen to other girls was positive	4.25	0.70
11.	I feel like I learned a lot about the challenges facing girls in our society	4.10	0.69
12.	The section on gratitude helped me to see what really matters in life	3.90	0.47
**Enjoyment of the program**			
1.	The HWB for Girls program was enjoyable	4.44	0.58
2.	The physical activity sessions were enjoyable	4.15	0.80
3.	The yoga activities were enjoyable	3.71	0.82
4.	I enjoyed being in a girls only zone and working with each other, not against each other	4.35	0.93

All items were measured on a 5-point Likert Scale (range from *Strongly Disagree to Strongly Agree*) with a maximum score of 5.0

### Preliminary efficacy

#### Mental health outcome

Our results indicate medium positive effects on four efficacy measures (evaluated using the Strengths and Difficulties Questionnaire), including total difficulties −2.0 [95% CI (−3.93 to −0.10), *d*=0.45], social health +1.7 [95% CI (0.25 to 3.16), *d*=0.50], problems with peers −1.0 [95% CI (−1.65 to −0.34), *d*=0.65] and internalizing −1.2 [95% CI (−2.28 to −0.12), *d*=0.47]. No effects resulted for mindfulness, self-compassion, or rumination.

#### Physical health outcomes

Small positive effects were observed for total PA (*d* = 0.24) and weekend PA (*d* = 0.32), and no effect was observed for weekday PA (*d* = 0.07).

#### Screen-time outcomes

No effects were observed for recreational screen-time levels for weekday, weekend, or total screen-time.

## Discussion

The primary objective of this study was to determine the feasibility of delivering the HWBG program in a secondary school setting as an elective subject for year 8 students. We also investigated the preliminary efficacy of the program on adolescent girls’ mental health, screen-time and physical activity levels. The feasibility of the program was confirmed with satisfactory retention, compliance, adherence, fidelity, and satisfaction ratings. The HWBG program was also successful in improving measures of mental and social health in girls participating in the program, in comparison to a control group.

### Feasibility of HWBG

The positive feedback and enjoyment scores reported by participants in the HWBG program implies that the program was well received by this group and feasible for use with year 8 girls in a secondary school setting. Participants reported the HWBG program to be enjoyable as indicated in the evaluation data (mean rating of 4.44 out of a possible 5.0). This is an important finding as enjoyment is one of the key mediators of engagement in school [26, 39]. The supportive environment created in HWBG was recognized by participants and rated highly: “I liked the fact that I had realised that all the girls in the class were feeling similar problems to mine and that I am not alone”. Evaluation data (mean score 4.35/5 and SD of 0.93) demonstrates that participants enjoyed the girls only program and working with each other. Enjoyment of physical activity is also observed to be a mediating factor contributing to increased participation in physical activity [65]. High levels of physical activity enjoyment during adolescence is also likely to lead to healthy lifelong physical activity behaviors [65]. Evaluation data from HWBG demonstrates high levels of enjoyment from participants regarding physical activity (mean score 4. 2 ± 0.8 out of 5). In addition, participants responded positively with a mean score of 4.1 out of 5, when asked if the physical activity sessions helped them to realize that they can do fitness activities.

Our results showed that physical activity and mindfulness were the two most highly rated program components. This is potentially attributed to the purposeful creation of an inclusive and socially supportive environment transcending competition, with a focus on lowering levels of judgment among participants. Elements of Acceptance and Commitment Therapy and mindfulness were used to navigate challenges and overcome barriers to physical activity through the willingness to experience negative emotions or thoughts whilst having the ability to engage in valued behaviors [52]. Self-compassion was utilized to create a supportive environment for adolescent girls in HWBG and entails three basic components of (a) self-kindness, (b) common humanity, and (c) mindfulness [70]. Self-compassion involves understanding one’s inadequacies and failures, utilizing a non-judgmental attitude toward the self and viewing these as part of a common human experience [73]. Improving levels of self-compassion in adolescents has been observed to promote positive body image in this group [31, 82]. Body image is recognized as a barrier to physical activity for adolescents [28], and individuals with higher levels of body image are more inclined to participate in physical activity than those adolescents with lower levels of body image [56]. The HWBG program had a positive impact on body image, with participants’ responses averaging 4.5 out of 5 when asked whether body image lessons helped them to realize that they do not need to be perfect. Qualitative data also support this finding: “It’s a great elective and I have learned lots of new skills and ways to see myself.”

An intervention program aimed at increasing physical activity levels of adolescent females known as “Girls on the Move” (GOTM) observed the importance of enjoyment of physical activity and social support for increasing levels of physical activity. Similar to the HWBG program, GOTM observed improved levels of physical activity enjoyment and social support in physical activity [81]. A unique element of the HWBG program was the use of ACT and mindfulness in addressing perceived barriers to physical activity for adolescent females including body image issues, time constraints [28], dislike of physical activity, and lack of perceived competence [107].

Mindfulness was ranked as the second highest component enjoyed by participants. Participants utilized the practice to increase their awareness of thoughts and emotions along with components of ACT such as acceptance, diffusion, being present, and commitment to values. Participants in HWBG learned to address and overcome barriers to participation in physical activity through the use of these therapies: “I mostly liked the physical activity because I never thought I could actually keep going in the 30 second workout, and that made me realise I could do more”. Only a small positive effect was observed for levels of total physical activity [+11183.7; 95% CI −3164.31–25531.79; *p*=0.124; *d*=0.24]—however, this could be interpreted as a positive outcome as a decline in physical activity levels was not observed (which is typical of adolescent girls). Furthermore, despite only small improvements in physical activity and mindfulness levels observed in the HWBG study the participants reported a very high level of enjoyment toward both of these program components.

Participants also commented that the HWBG program was fun and exciting. “What I enjoyed about HWBG was the practical lessons because it made me enjoy doing sport and I made a lot of new friends. I loved the chocolate meditation, but most of all just the whole program, it was really fun and exciting.” Research suggests that fun and excitement are elements that have contributed to the positive effects observed for the social and psychological health improvement levels of the participants at a 6-month follow-up [57, 63, 98].

### Preliminary efficacy

Medium positive effects were observed for internalizing and total difficulties among the participants in the HWBG program. Internalizing refers to symptoms of depression, anxiety, hypersensitivity, and worry problems [101]. In general, girls report more internalizing problems than boys, although these differences are small, according to a meta-analysis review [19]. Among youth, internalizing problems include lack of joy, impaired self-worth, disrupted sleep, and appetite patterns which negatively impact the everyday lives and health status for youths. Increased risk of suicide and self-harm are also associated with internalizing symptoms [87]. Warm, caring environments where both peers and adults are supportive, contribute to protection against increased levels of internalizing among adolescents [69]. Given that approximately 20% of adolescents worldwide have a diagnosed mental health illness [78], this program may provide a possible strategy for addressing psychological health issues within this high-risk population.

The Penn Resiliency Program (PRP) is a cognitive behavioral therapy program designed to reduce internalizing in adolescents. The PRP has demonstrated small but reliable reductions in depressive symptoms in adolescents [15]), but has reported no intervention effects on internalizing levels [23]. Research has shown that not all people suffering depressive and anxiety disorders respond well to cognitive behavior therapy [10], and there are high rates of relapse following treatment [14]—warranting efforts to develop and trial additional methods to address these symptoms. MoodGym is another wellbeing intervention aimed at reducing depressive symptoms in adolescent girls and is a self-directed program that is completed online. MoodGym reported benefits on self-reported symptoms of depression, but observed low completion rates—suggesting challenges with adherence levels to online programs [76].

Mindfulness practices were used consistently in the HWBG program aiming to develop a flexibility of thinking and the ability to become an observer of the mind without getting “caught up” in negative thought patterns. Participants reported in the evaluation data that the HWBG program had a positive impact on their levels of emotional health: “Health and well-being has helped me with my everyday life. It has made me confident with who I am. I am super happy that this course has changed the way I think, because I have been in a dark headspace for a while, which definitely isn’t good”. Although initial findings have been positive, there is a need for more rigorous evaluation of mindfulness training in adolescent populations [84].

Evidence demonstrates the importance of managing emotional health problems early in adolescence and shifting from treatment to prevention, as there are implications for school attendance, self-esteem, and prosocial behavior [67, 104]. As emotional ill health can increase in this stage of development, early adolescence is an opportune time for interventions of a preventative nature. Mindfulness-based interventions (MBI) may directly reduce stress, anxiety, and depression, whilst increasing psychological well-being [48]. This is supported by a recent meta-analysis indicating significant positive effects of MBI across randomized controlled trials with active control groups on mindfulness (*d*= .42), depression (*d*= .47), and anxiety/stress (*d*= .18) [27]. Furthermore, school-based mindfulness programs have shown to prevent internalizing problems in adolescents [75].

Medium positive effects were observed for levels of social health and problems with peers among the participants in HWBG. This is a promising result as healthy interpersonal relationships and social support are integral to well-being, and levels of physical and mental health [50, 74, 97]. Social isolation and loneliness however are associated with a broad range of mental health difficulties in adolescents (e.g., suicidal ideation, anxiety, depression, substance misuse, self-injury) [44, 46, 61, 90]. The HWBG program has a strong focus on creating a supportive learning environment where participants have the autonomy to discuss issues that are of importance to them without fear of judgment from others. This element may have contributed to improvements in these measures of social health and problems with peers. Connecting in a caring environment and discussing common concerns and challenges in their lives may have also enhanced their sense of competency [33].

Participants enjoyed completing the program designed for girls only and working collaboratively in this girls’ only zone. Feasibility results observe a mean result of 4.4 out of a possible 5.0 when asked whether they enjoyed working together and qualitative data reports: “I enjoyed being able to talk to other girls my age who normally I wouldn’t really talk to”. “I loved being with a group of girls only and the feeling of not being judged”. High levels of reported enjoyment of HWBG from the participants and improvements in social health indicate that strong connections were established among the participants which contributed to improved levels of well-being [83] and positive outcomes in the physical activity setting [17]. Our positive findings with regard to social health will add to the limited literature in this field. Strategies that focus on the promotion of good social health and social connectedness may offer a level of protection for young people from loneliness and contribute to improved levels of well-being [22, 58, 83].

### Strengths and limitations

The strengths of this study include the novel intervention design, adherence to CONSORT guidelines, and the assessment of outcome using assessors who were blinded to group allocation. However, there are some limitations that should be acknowledged. First, the study included a small convenience sample in one secondary school. Second, a member of the research team, who was also a teacher at the school, delivered the program. There is inherent risk of bias associated with this feasibility study as the teacher, who was also a member of the research team, designed and delivered the program and was also involved in the evaluation of the program. And although conducting a single site feasibility study is a limitation, it is an important and necessary step in testing program content and delivery under “ideal conditions” prior to large-scale implementation. Given that this study was designed to establish feasibility and preliminary efficacy of the program for use with adolescent girls in the school setting, using a member of the research team for delivery was a necessary step. Third, physical activity was assessed using pedometers, not accelerometers which are considered more appropriate for the evaluation of school-based physical activity interventions. Future studies are encouraged to use accelerometers to provide an objective measure of physical activity intensity and duration [60]. To establish effectiveness and scalability, further large-scale trials are needed whereby teachers in the school will be trained utilizing a “train the teacher model” in order to reach more schools in a variety of cohorts without researcher assistance [2].

### Recommendations

Developing a targeted intervention for a population group who have been recognized as “at risk” of developing a range of short- and long-term health problems (associated with low levels of physical activity, well-being, mental health, and screen-time behaviors) may have a profound impact. This feasibility study may have positive short- and long-term benefits for adolescent girls participating in the program, but future large-scale program implementation and dissemination in secondary schools is needed for wide scale impact. Based on the results of this study, we would recommend the program be evaluated in a cluster FRCT involving multiple schools. In this phase of research, the program should be delivered by teachers who have received training, rather than members of the research team. Progression to a full-powered trial will be dependent upon the feasibility and preliminary efficacy of the “train-the-teacher” delivery model.

HWBG is a unique program that is the first of its kind to combine well-being, mental health, physical activity, mindfulness, and screen-time behaviors into one program with a theoretical grounding in Self-Determination Theory, supported by elements of Acceptance and Commitment Therapy. Training educators in mindfulness would be highly recommended for effective delivery of this intervention, as practicing mindfulness within an educational setting provides a potentially feasible and effective strategy for improving the psychological health of young people [63].

## Conclusion

Our findings suggest that the HWBG program is feasible for use in one school setting, based on high levels of retention, satisfaction, and fidelity. In addition, our study has provided preliminary evidence that the HWBG program may facilitate improvements in psychological and social health outcomes in adolescent girls. Based on the results of this study, we would recommend the program be evaluated in a cluster FRCT involving multiple schools before progressing to a large-scale effectiveness trial.

## Data Availability

The datasets during and/or analyzed during the current study available from the corresponding author on reasonable request.

## References

[1] Active Healthy Kids Australia. Muscular fitness: it’s time for a jump start. The 2018 active healthy kids Australia report card on physical activity for children and young people. Adelaide: Active Healthy Kids Australia; 2018. 10.25954/5b862301479a1.

[2] Adams J (2006). Trends in physical activity and inactivity amongst US 14–18 year olds by gender, school grade and race, 1993–2003: evidence from the youth risk behavior survey. BioMed Central Public Health.

[3] Amarson E, Craighead W (2009). Prevention of depression among Icelandic adolescents. Behav Res Ther.

[4] Anderman EM (2002). School effects on psychological outcomes during adolescence. J Educ Psychol.

[5] Aubert S, Barnes JD, Abdeta C, Nader PA, Adeniyi AF, Aguilar-Farias N, Cardon G (2018). Global matrix 3.0 physical activity report card grades for children and youth: results and analysis from 49 countries. J Phys Act Health.

[6] Australian Bureau of Statistics (2015). National health survey: first results, 2014–2015 (4364.0.55.001).

[7] Bailey A, Hetrick S, Rosenbaum S, Purcell R, Parker A (2018). Treating depression with physical activity in adolescents and young adults: a systematic review and meta-analysis of randomised controlled trials. Psychol Med.

[8] Babic MJ, Smith JJ, Morgan PJ, Lonsdale C, Plotnikoff R, Eather N, Lubans DR. Intervention to reduce recreational screen-time in adolescents: outcomes and mediators from the 'Switch-Off 4 Healthy Minds' (S4HM) cluster randomised controlled trial. Prev Med. 2016;91:50–7. 10.1016/i.ypmed.2016.07.014. 10.1016/j.ypmed.2016.07.014 27471018

[9] Barber BK, Schluterman JM (2008). Connectedness in the lives of children and adolescents: a call for greater conceptual clarity. J Adolesc Health.

[10] Barlow DH, Gormon JM, Shear MK, Woods SW (2000). Cognitive-behavioral therapy, imipramine, or their combination for panic disorder: a randomized controlled trial. JAMA.

[11] Bell SL, Audrey S, Gunnell D, Cooper A, Campbell R (2019). The relationship between physical activity, mental wellbeing and symptoms of mental health disorder in adolescents: a cohort study. Int J Behav Nutr Phys Act.

[12] Biddle S, Ciaccioni S, Thomas G, Vergeer I. Physical activity and mental health in children and adolescents: an updated review of reviews and an analysis of causality. Psychol Sport Exerc. 2018. 10.1016/j.psychsport.2018.08.011.

[13] Bond L, Butler H, Thomas L (2007). Social and school connectedness in early secondary school as predictors of late teenage substance use, mental health, and academic outcomes. J Adolesc Health.

[14] Brown TA, Barlow DH (1995). Long-term outcome in cognitive-behavioral treatment of panic disorder: clinical predictors and alternative strategies for assessment. J Consult Clin Psychol.

[15] Brunwasser SM, Gillham JE, Kim ES (2009). A meta-analytic review of the Penn Resiliency Program’s effect on depressive symptoms. J Consult Clin Psychol.

[16] Bullot A, Cave L, Fildes J, Hall S, Plummer J. Mission Australia’s Youth Survey Report, Mission Australia; 2017.

[17] Camacho-Minano MJ, LaVoi NM, Barr-Anderson DJ (2011). Interventions to promote physical activity among young and adolescent girls: a systematic review. Health Educ Res.

[18] Catalano R, Berglund M, Ryan J, Lonczak H, Hawkins J. Positive youth development in the United States: research findings on evaluations of positive youth development programs. Prev Treat. 2002;5. 10.1037//1522-3736.5.1.515a.

[19] Chaplin TM, Aldao A (2013). Gender differences in emotion expression in children: a meta-analytic review. Psychol Bull.

[20] Childline (2018). Social media could be responsible for rise in lonely children, childline warns., from Independent.

[21] Cohen J (1988). Statistical power analysis for the behavioral sciences.

[22] Cruwys T, Dingle G, Haslam S, Jetten J, Morton T (2013). Social group memberships protect against future depression, alleviate depression symptoms and prevent depression relapse. Soc Sci Med.

[23] Cutuli JJ, Gillham JE, Chaplin TM, Reivich KJ, Seligman M, Gallop RJ (2013). Preventing adolescents’ externalizing and internalizing symptoms: effects of the Penn Resiliency Program. Int J Emot Educ.

[24] Deci EL, Ryan RM (2012). Self-determination theory. Handbook of theories of social psychology.

[25] Diener E, Wirtz D, Tov W, Kim-Prieto C, Choi DW, Oishi S, Biswas-Diener R (2010). New well-being measures: short scales to assess flourishing and positive and negative feelings. Social Indicator Res.

[26] Dishman R, Motl R, Saunders R, Felton G, Ward D, Dowda M, Pate R (2005). Enjoyment mediates effects of a school-based physical-activity intervention. Med Sci Sports Exerc.

[27] Dunning D, Griffiths K, Kuyken W, Crane C, Foulkes L, Parker J, Dalgleish T (2018). The effects of mindfulness-based interventions on cognition and mental health in children and adolescents: a meta-analysis of randomised controlled trials.

[28] Dwyer J, Allison K, Goldenberg E, Fein A, Yoshida K, Boutilier M (2006). Adolescent girls’ perceived barriers to participation in physical activity. Adolescence.

[29] Eldridge SM, Chan CL, Campbell MJ, Bond CM, Hopewell S, Thabane L, et al. CONSORT 2010 statement: extension to randomised pilot and feasibility trials. Br Med J. 2016;355. 10.1136/bmj.i5239 PMC5076380 27777223

[30] Erskine H, Baxter A, Patton G, Moffitt T, Patel V, Whiteford HA, Scott J (2017). The global coverage of prevalence data for mental disorders in children and adolescents. Epidemiol Psychiatr Sci.

[31] Ferreira C, Pinto-Gouveia J, Duarte C (2013). Self-compassion in the face of shame and body image dissatisfaction: implications for eating disorders. Eat Behav.

[32] Finch LR, Hargrave J, Nichols, & A Van Vliet. (2014). Measure what you treasure: wellbeing and young people. How it can be measured and what the data tell us. New Philanthropy Capital.

[33] Fredricks J, Alfeld C, Eccles J (2010). Developing and fostering passion in academic and nonacademic domains. Gifted Child Quart - GIFTED CHILD QUART.

[34] Galderisi S, Heinz A, Kastrup M, Beezhold J, Sartorius N (2015). Toward a new definition of mental health. World Psychiatry.

[35] Girlguiding (2015). Girls’ attitude survey.

[36] Goodman R (2001). Psychometric properties of the strengths and difficulties questionnaire. J Am Acad Child Adolesc Psychiatry.

[37] Goodman R, Meltzer H, Bailey V (2003). The Strengths and Difficulties Questionnaire: a pilot study on the validity of the self-report version. Int Rev Psychiatry.

[38] Greco LA, Baer RA, Smith GT (2011). Assessing mindfulness in children and adolescents: development and validation of the Child and Adolescent Mindfulness Measure (CAMM). Psychol Assess.

[39] Hagenauer G, Hascher T. Early adolescents enjoyment experienced in learning situations at school and its relation to student achievement. J Educ Train Stud. 2014;2(2).

[40] Haidt J (2017). The unwisest idea on campus: commentary on Lilienfield. Perspect Psychol Sci.

[41] Hall-Lande JA, Eisenberg ME, Christensen SL, Neumark- Sztainer D (2007). Social isolation, psychological health and protective factors in adolescence. Adolescence.

[42] Hardy LL, Booth ML, Okely AD (2007). The reliability of the adolescent sedentary activity questionnaire (ASAQ). Prev Med.

[43] Hartas, D. (2019). The social context of adolescent mental health and wellbeing: parents, friends and social media (Publication no. 10.1080/02671522.2019.1697734). from Routledge: Taylor and Francis Group

[44] Hassed C (2008). The essence of health.

[45] Hayes SC (2004). Acceptance and commitment therapy, relational frame theory, and the third wave of behavioral and cognitive therapies. Behav Ther.

[46] Heinrich L, Gullone E (2006). The clinical significance of loneliness: a literature review. Clin Pyschol Rev.

[47] Hoare E, Milton K, Foster C, Allender S (2016). The associations between sedentary behaviour and mental health among adolescents: a systematic review. Int J Behav Nutr Phys Act.

[48] Hofmann SG, Gómez AF (2017). Mindfulness-based interventions for anxiety and depression. Psychiatr Clin North Am.

[49] International Positive Education Network. (2017). The state of positive education (Publication no. https://www.worldgovernmentsummit.org/api/publications/document/8f647dc4-e97c-6578-b2f8-ff0000a7ddb6). Retrieved 20th November 2019

[50] Jose PE, Lim BT (2014). Social connectedness predicts lower loneliness and depressive symptoms over time in adolescents. Open J Depress.

[51] Jose PE, Ryan N, Pryor J (2012). Does social connectedness promote a greater sense of wellbeing in adolescence over time?. J Res Adolesc.

[52] Juarascio A, Shaw J, Forman E, Timko CA, Herbert J, Butryn M, Lowe M (2013). Acceptance and commitment therapy as a novel treatment for eating disorders: an initial test of efficacy and mediation. Behav Modif.

[53] Karcher MJ, Finn L (2005). How connectedness contributes to experimental smoking among rural youth: developmental and ecological analyses. J Prim Prev.

[54] Karcher MJ, Sass D (2010). A multicultural assessment of adolescent connectedness: testing measurement invariance across gender and ethnicity. J Couns Psychol.

[55] Kelly YA, Zilanawala C, Booker A, Sacker A (2018). Social media use and adolescent mental health: findings from the UK millenium cohort study. E Clin Med.

[56] Kirkcaldy BD, Shephard RJ, Siefen RG (2002). The relationship between physical activity and self-image and problem behaviour among adolescents. Soc Psychiatry Psychiatr Epidemiol.

[57] Laird Y, Fawkner S, Niven A. A grounded theory of how social support influences physical activity in adolescent girls. Int J Qualit Stud Health Wellbeing. 2018;13. 10.1080/17482631.2018.1435099. 10.1080/17482631.2018.1435099 PMC5814762 29405881

[58] Lim M, Eres R, Peck C (2019). The young Australian loneliness survey. Understanding loneliness in adolescence and young adulthood.

[59] Liu M, Wu L, Yao S (2016). Dose-response association of screen time-based sedentary behaviour in children and adolescents and depression: a metaanalysis of observational studies. Br J Sports Med.

[60] Lubans DR, Plotnikoff RC, Miller A, Scott JJ, Thompson D, Tudor-Locke C. Using pedometers for measuring and increasing physical activity in children and adolescents: the next step. Am J Lifestyle Med. 2014. 10.1177/1559827614537774.

[61] Mahon N, Yarcheski A, Yarcheski T, Cannella B, Hanks M (2006). A meta-analytic study of predictors for loneliness during adolescence. Nurs Res.

[62] Markland D, Tobin V (2004). A modification to the behavioural regulation in exercise questionnaire to include an assessment of amotivation. J Sport Exerc Psychol.

[63] McGill J, Adler-Baeder F. Exploring the Link between mindfulness and relationship quality: direct and indirect pathways. J Marital Fam Ther. 2016;46(4). 10.1111/jmft.12412. 10.1111/jmft.12412 31630430

[64] McNamara E, Hudson Z, Taylor SJC (2010). Measuring activity levels of young people: the validity of pedometers. Br Med Bull.

[65] Michael SL, Coffield E, Lee SM, Fulton JE (2016). Variety, enjoyment, and physical activity participation among high school students. J Phys Act Health.

[66] Monshouer K, Van Poppel M, Vollebergh W, Ten Have M, Kemper H (2013). Possible mechanisms explaining the association between physical activity and mental health: findings from the 2001 Dutch Health Behaviour in School-Aged Children Survey. Clin Psychol Sci.

[67] Morgan A, Currie C, Due P, Gabhain SN, Rasmussen M, Samdal O, Smith R (2008). Mental wellbeing in school-aged children in Europe: associations with social cohesion and socioeconomic circumstances. Social Cohesion for Mental Well-Being Among Adolescents.

[68] Muris P, Meesters C, Fijen P (2003). The Self-Perception Profile for Children: further evidence for its factor structure, reliability, and validity. Personal Individ Differ.

[69] National Preventative Health Task Force. Australia: The Healthiest Country by 2020 -National Preventative Health Strategy - the roadmap for action. Commonwealth of Australia. 2009. ISBN: 1-74186-919-6.

[70] Neff K (2003). Self-Compassion: an alternative conceptualization of a healthy attitude toward oneself. Self Identity.

[71] Neff K (2003). Development and validation of a scale to measure self-compassion. Self Identity.

[72] Neff K (2016). The self-compassion scale is a valid and theoretically coherent measure of self-compassion. Mindfulness.

[73] Neff K, Germer C (2017). Self-compassion and psychological wellbeing. Oxford handbook of compassion science. Chapter 27.

[74] Norrish JM, Williams P, O'Connor M, Robinson J (2013). An applied framework for positive education. Int J Wellbeing.

[75] Novak M, Mihic J (2018). Prevention of internalised problems of children and youth in academic setting.

[76] O'Kearney R, Kang K, Christensen H, Griffiths K (2009). A controlled trial of a school-based Internet program fro reducing depressive symptoms in adolescent girls.

[77] Owen MB, Curry WB, Kerner C, Newson L, Fairclough SJ (2017). The effectiveness of school-based physical activity interventions for adolescent girls: a systematic review and meta-analysis. Prev Med.

[78] Patton G, Coffey C, Cappa C, Currie D, Riley L, Gore F, Ferguson J (2012). Health of the world’s adolescents: a synthesis of internationally comparable data. Lancet.

[79] Pearson N, Braithwaite R, Biddle S. The effectiveness of interventions to increase physical activity among adolescent girls: a meta-analysis. Acad Pediatr. 2015;15(1). 10.1016/j.acap.2014.08.009 25441655

[80] Pittman LD, Richmond A (2007). Academic and psychological functioning in late adolescence: the importance of school belonging. J Exp Educ.

[81] Robbins LB, Wen F, Ling J (2019). Mediators of physical activity behavior change in the “Girls on the Move” intervention. Nurs Res.

[82] Rodgers RF, Donovan E, Cousineau T, Yates K, McGowan K, Cook E, et al. BodiMojo: efficacy of a mobile-based intervention in improving body image and self-compassion among adolescents. J Youth Adolesc. 2018. 10.1007/s10964-017-0804-3. 10.1007/s10964-017-0804-3 29349593

[83] Roffey S (2011). Enhancing connectedness in Australian children and young people. Asian J Couns.

[84] Salmoirago-Blotcher E, Druker S, Frisard C, Dunsiger SI, Crawford S, Meleo-Meyer F (2018). Integrating mindfulness training in school health education to promote healthy behaviours in adolescents: feasibility and preliminary effects on exercise and dietary habits. Prev Med Rep.

[85] Schneider PL, Crouter SE, Lukajic O (2003). Accuracy and reliability of 10 pedometers for measuring steps over a 400-m walk. Med Sci Sports Exerc.

[86] Schönfeld P, Brailovskaia J, Margraf J (2017). Positive and negative mental health across the lifespan: a cross-cultural comparison. Int J Clin Health Psychol: IJCHP.

[87] Schulte-Körne G (2016). Mental health problems in a school setting in children and adolescents. Dtsch Arztebl Int.

[88] Seligman M (2011). Flourish: a new understanding of happiness and wellbeing and how to achieve them.

[89] Shensa ACG, Escobar-Viera JE, Sidani ND, Bowman MP, Marshal, & Primack, B. A. (2017). Problematic social media use and depressive symptoms among U. S young adults: a nationally- representative study. Soc Sci Med.

[90] Shevlin M, Murphy J, Mallet J, Stringer M, Murphy J (2013). Adolescent loneliness and psychiatric morbidity in Northern Ireland. Br J Clin Psychol.

[91] Shochet IM, Dadds MR, Ham D, Montague R (2006). School connectedness is an underemphasized parameter in adolescent mental health: results of a community prediction study. J Clin Child Adolesc Psychol.

[92] Stroud CB, Fitts J. Rumination in early adolescent girls: interactive contributions of mother–adolescent relationship quality and maternal coping suggestions. J Clin Child Adolesc Psychol. 2015:1–12. 10.1080/15374416.2015.1094737. 10.1080/15374416.2015.1094737 26645734

[93] Tremblay MS, LeBlanc AG, Kho ME, Saunders TJ, Larouche R, Colley RC, et al. Systematic review of sedentary behaviour and health indicators in school-aged children and youth. Int J Behav Nutr Phys Act. 2011;8(98). 10.1186/1479-5868-8-98 PMC3186735 21936895

[94] Treynor W, Gonzalez R, Nolen-Hoeksema S (2003). Rumination reconsidered: a psychometric analysis. Cognit Ther Res.

[95] Twenge JM (2017). Have smartphones destroyed a generation?.

[96] Twenge JM, Joiner TE, Rogers ML. Increases in depressive symptoms, suicide-related outcomes, and suicide rates among U.S. adolescents after 2010 and links to increased new media screen time. Clin Psychol Sci. 2017.

[97] Uchino BN, Cacioppo JT, Kiecolt-Glaser JK. The relationship between social support and physiological processes: a review with emphasis on underlying mechanisms and implications for health. Psychol Bull. 1996:488–531. 10.1037/0033-2909.119.3.488. 10.1037/0033-2909.119.3.488 8668748

[98] Vaquero-Solís M, Gallego DI, Tapia-Serrano MA, Pulido JJ, Sánchez-Miguel PA (2020). School-based physical activity interventions in children and adolescents: a systematic review.

[99] Waters L (2011). A review of school-based positive psychology interventions. Aust Educ Dev Psychol.

[100] Weare K. Editorial: child and adolescent mental health in schools. Child Adolesc Mental Health. 2015;20. 10.1111/camh.12044 32680386

[101] Weeks M, Ploubidis GB, Cairney J (2016). Developmental pathways linking childhood and adolescent internalizing, externalizing, academic competence, and adolescent depression. J Adolesc Health.

[102] Weisz JR, McCarty CA, Valeri SM. Effects of psychotherapy for depression in children and adolescents: a meta-analysis. Psychol Bull. 2006;(1):132–49. 10.1037/0033-2909.132.1.132 PMC2150594 16435960

[103] Werner EE, Smith RS (2001). Journeys from childhood to midlife: risk, resilience, and recovery.

[104] World Health Organisation. Adolescent mental health: Mapping actions of non-governmental organizations and other international development organizations. Geneva. 2012. 9789241503648.

[105] World Health Organisation (2014). Global recommendations on physical activity for health.

[106] World Health Organization (2019). Adolescent mental health.

[107] Zaragoza J, Generelo E, Julián JA, Abarca-Sos A (2011). Barriers to adolescent girls’ participation in physical activity defined by physical activity levels. J Sports Med Phys Fitness.

[108] Zhai L, Zhang D (2015). Sedentary behaviour and the risk of depression: a meta-analysis. Br J Sports Med.

